# Lithobius (Monotarsobius) zhangi sp. n., a new species from Eastern China (Chilopoda, Lithobiomorpha, Lithobiidae)

**DOI:** 10.3897/zookeys.459.8169

**Published:** 2014-12-01

**Authors:** Huiqin Ma, Sujian Pei, Xiaojie Hou, Tiegang Zhu

**Affiliations:** 1 Scientific Research Office, Hengshui University, Hengshui, Hebei 053000, P. R. China; 2 Department of Life Sciences, Hengshui University, Hengshui, Hebei 053000, P. R. China

**Keywords:** Lithobiidae, Lithobius (Monotarsobius) zhangi, Shandong Province, China, identification key

## Abstract

Lithobius (Monotarsobius) zhangi **sp. n.** (Lithobiomorpha: Lithobiidae), recently discovered from Nanshan Park, Yantai City, Shandong Province, and Wuyishan County, Nanping City, Fujian Province, from China, is described. Morphologically it resembles Lithobius (Monotarsobius) songi Pei, Ma, Shi, Wu, Zhou, 2011 from Province Hebei, China, but can be readily distinguished from the latter by antennae composed of 15+15–19+19 articles versus 19+19–21+21 articles, terminal claw of female gonopods inner tooth broader than the outer vs dorsal and ventral tooth about same in size, ventral plectrotaxy 01320, dorsal plectrotaxy 10210 in the 14th legs, 01210 and 10200 respectively in Lithobius (Monotarsobius) songi. A key to the Lithobius (Monotarsobius) species of China and Korea is presented.

## Introduction

The centipede subgenus Lithobius (Monotarsobius) Verhoeff, 1905 (Lithobiomorpha: Lithobiidae) is characterized by the presence of fused tarsi of legs 1–13 and antennal articles fixed at 20 or thereabouts (Eason 1992), this subgenus comprises 114 species known from Asia, Europe, and North Africa (Pocock 1895; Trotzina 1895; Attems 1901, 1904; Dobroruka 1960, 1979; Zalesskaja 1978; Farzalieva and Zalesskaja 2002; Farzalieva 2006; Zapparoli 2006; Zapparoli and Edgecombe 2011; Dányi and Tuf 2012).

Lithobiomorph centipedes of China are poorly known as only sixty-nine species and subspecies are hitherto known from the country (Attems 1938, 1953; Takakuwa 1939, 1940; Takakuwa and Takashima 1949; Chamberlin and Wang 1952; Wang 1959, 1963; Zalesskaja 1978; Wang and Mauriès 1996; Zhang 1996; Eason 1997, 1997; Chao 2005; Zapparoli 2006; Ma et al. 2007a, b, c; 2008a, b, c, d; Ma et al. 2009a, b; 2012a, b; 2013; 2014; Pei et al. 2010; 2011a, b). The subgenus Lithobius (Monotarsobius) is among the poorly studied taxa of China, with only ten species being up to now registered from its territory. None of them has hitherto been documented from Shandong Province. Herewith we describe a new species recently found in Shandong and Fujian Provinces.

## Methods

All specimens were hand-collected under leaf litter or stones. The material was examined with the aid of a Motic-C microscope, made in China. The colour description is based on specimens in 75% ethanol, and body length is measured from anterior margin of the cephalic fig to posterior end of postpedal tergite. Type specimens are preserved in 75% ethanol and deposited in the department of Life Sciences, Hengshui University, Hengshui, China. The terminology of the external anatomy follows Bonato et al. (2010).

The following abbreviations are used in the text and the tables: T, TT = tergite, tergites; S, SS = sternite, sternites; C = coxa, Tr = trochanter, P = prefemur, F = femur, Ti = tibia, a = anterior, m = median, p = posterior.

## Taxonomic part Lithobiidae Newport, 1844

### Lithobius (Monotarsobius) zhangi sp. n.

Taxon classificationAnimaliaLithobiomorphaLithobiidae

http://zoobank.org/32726748-44E8-452C-A4FC-C461A084B73B

Figs 1 –6

#### Material examined.

**Figures 1–6.**
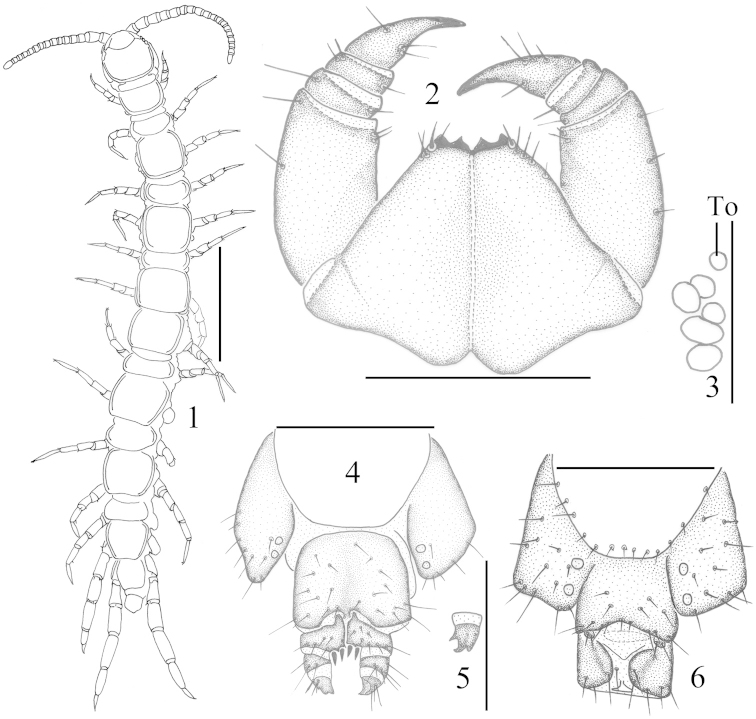
Lithobius (Monotarsobius) zhangi sp. n., **1–5** holotype, female: **1** habitus, dorsal view, scale bar 1 mm; **2** forcipular segment, ventral view, scale bar 500 μm; **3** ocelli and Tömösváry’s organ (To), lateral view, scale bar 250 μm; **4** posterior segments and gonopods, ventral view, scale bar 500 µm; **5** terminal claw of right gonopod, dorsal view, scale bar 250 µm; **6** paratype, male: posterior segments and gonopods, ventral view, scale bar 500 µm.

**Holotype.** ♀ (Figure 1), body length 8.0 mm, cephalic fig 0.5 mm long, 0.5 mm broad, Nanshan Park, Yantai City, Shandong Province, 37°05'N, 121°04'E, 27 m, 5 July 2005, leg. Huiqin Ma. **Paratypes.** 2 ♀♀, same data as holotype.

**Other material.** 15 ♀♀, 2 ♂♂, Wuyishan County, Nanping City, Fujian Province, 27°43'N 118°01'E, 238 m, 10 August 2010, leg. Feng Zhang and Huiqin Ma.

#### Etymology.

The specific name is a patronym in honor of the myriapodologist Professor Chongzhou Zhang, Academician at the Chinese Academy of Sciences.

#### Diagnosis.

A Lithobius (Monotarsobius) species with body length 7.0–8.0 mm, antennae composed of 15–19 articles; 5–6 ocelli on each side, arranged in 2 irregular rows, the terminal ocellus comparatively large; Tömösváry’s organ moderately small, slightly smaller than adjoining ocelli; 2+2 coxosternal teeth; porodonts moderately slender, posterolateral to the most lateral teeth; posterior angles of all tergites without triangular projections; coxal pores 1222, oval to round; female gonopods with 2+2 small, coniform spurs; terminal claw of the third article tridentate; male gonopods short and small, with 1 long seta on the terminal segment.

#### Description.

Body length: 7.0–8.0 mm, cephalic fig 0.5–0.6 mm long, 0.5–0.6 mm wide.

Colour: basal antennal articles lavender, the 7–8 article gradually turning to yellow-brown, distalmost article yellow-brown; tergites pale brown to chestnut-brown; cephalic fig, TT1, 14 and 15 yellow-brown; pleural region pale grey to lavender; sternites pale grey to gray; distal part of forcipules brown, basal and proximal parts of forcipules, forcipular coxosternite and SS 14 and 15 pale yellow-brown with greyish hue; all legs lavender, the distal of every article of all legs slightly dark, the tarsus of all legs yellow-brown.

Antennae: 15–19 articles (Figure 1); basal article slightly longer than wide, second one markedly longer than wide, following articles gradually shortening, distal article up to 2.0–2.5 times as long as wide. Abundant setae on the antennal surface, less so on the basal articles, gradually increasing in density to about sixth article, then more or less constant.

Cephalic fig smooth, convex, width approximately equal to length; tiny setae emerging from pores scattered very sparsely over the whole surface; frontal marginal ridge with shallow anterior median furrow; short to long setae scattered along the marginal ridge of the cephalic fig; lateral marginal ridge discontinuous, posterior marginal ridge moderately broader, straight or slightly bulging.

Five–six oval to rounded ocelli on each side (Figure 3) in two irregular rows; the terminal ocellus comparatively large; other ocelli about equal in size apart the ocelli adjoining to the ventral; all ocelli domed, translucent, usually darkly pigmented.

Tömösváry’s organ situated at the anterolateral margin of the cephalic fig, slightly smaller than the adjoining ocelli and lying well apart from them (Figure 3-To).

Coxosternite subtrapezoidal (Figure 2), anterior margin narrow; median diastema moderately deep, V-shaped; anterior margin with 2+2 teeth; porodonts slender, lying posterolateral to the most lateral teeth (Figure 4); some long setae scattered on the ventral side of coxosternite.

All tergites smooth, without wrinkles, backside slightly hunched; T 1 posterolaterally narrower than anterolaterally, generally trapeziform, narrower than T 3 and the cephalic fig, the cephalic fig slightly wider than T 3 or equal to T 3; posterior margin of T 1 straight or slightly convex, its posterior marginal ridge continuous; posterior margin of TT 3, 5, 8, 10, 12 and 14 shallow concave, posterior marginal ridge of TT 3, 5, 8, 10 and 12 discontinuous; all posterior angles generally rounded, without triangular projections; lateral marginal ridge of all tergites continuous (Figure 1); tiny setae scattered very sparsely over the surface.

Posterior side of sternites narrower than the anterior one, generally trapeziform, comparatively smooth, setae emerging from pores scattered very sparsely on the surface, slightly thicker setae on the surface of the anterior part of each sternite; A pair of longer setae approximately symmetrical on the surface of both the anterior and the posterior part of each sternite; 2–3 longer setae on both anterior lateral borders, 1–2 comparatively long setae scattered sparsely on posterior margin of sternites.

Legs strong, tarsal articulation not defined on legs 1–13, tarsal articulation well defined on legs 14–15; all legs with fairly long curved claws; anterior and posterior accessory spines on legs 1–14; anterior accessory spine moderately long and slender, the posterior one slightly strong; the anterior accessory spines form relatively large angles with the pretarsus, the posterior accessory spines form relatively small angles with the pretarsus; no anterior accessory spines on legs 15. Short to comparatively long setae scattered very sparsely over the surface of all segments of all legs, more setae scattered on the surface of tarsus, slightly thick setae arranged in a row on the ventral side of tarsus; legs 14 and 15 absence of secondary sexual characters on femur or tibia, obvious thicker and stronger than other legs, tarsus 1 about 3.3–4.5 times as long as wide, tarsus 2 about 65%–82% the length of tarsus on legs 15. Leg plectrotaxy as in Table 1.

Coxal pores 1222, round or slightly ovate, coxal pore field in a relatively flat surface.

Female S 15 anterolaterally broader than posterolaterally, generally trapeziform, posteromedially straight, generally yellow-brown; short to long setae scattered sparsely on the surface and the lateral margin, 2 longer setae on posterior lateral borders; sternite of genital segment usually well chitinised, wider than long; relatively long setae scattered over the ventral surface of the genital segment, few setae near S 15, regularly fringed with longer setae along the posterior margin; posterior margin of genital sternite deeply concave between the condyles of gonopods, except for a small, median approximately triangular bulge. Gonopods: first article fairly broad, bearing 7–8 long setae arranged in three irregular rows; 2+2 moderately small, blunt, coniform spurs, inner spur slightly smaller than the outer (Figure 4); second article with 3–4 rather long setae, arranged in two irregular rows on the ventral side, third article with 2 comparatively long setae lying on the ventral side, terminal claw tridentate, the inner broader than the outer (Figure 5).

Male S 15 posterolaterally narrower than anterolaterally, generally trapeziform, posteromedially straight, sparsely covered with short to long setae; the sternite of the genital segment wider than long, usually well sclerotised. Posterior margin quite deeply concave between the gonopods, without a medial bulge; comparatively long setae evenly scattered on the ventral surface of the genital segment, few setae near S 15, gonopod short, consisting of a small bulge, with two long setae, apically slightly sclerotised (Figure 6).

#### Habitat.

The specimens were collected in a *Larix* forest. The species inhabits moderately moist habitats under roadside stones and forest floor.

#### Remarks.

The new species is morphologically close to Lithobius (Monotarsobius) songi Pei, Ma, Shi, Wu, Zhou, 2011 from Province Hebei, China, with which it shares the following traits: 2+2 coxosternal teeth, 2+2 spurs of female gonopods and 1222 coxal pores, the terminal claw of the female gonopods tridentate. It can however be distinguished from the latter by antennae composed of 15+15–19+19 articles versus 19+19–21+21 articles, terminal claw of female gonopods inner tooth broader than the outer vs dorsal and ventral tooth about same in size, ventral plectrotaxy 01320, dorsal plectrotaxy 10210 in the 14th legs, 01210 respectively 10200 in Lithobius (Monotarsobius) songi. The new species is morphologically close to Lithobius (Monotarsobius) dziadoszi Matic, 1970, from Korea, with which it shares the following traits: antennae composed of 15–20 articles, 2+2 coxosternal teeth, 2+2 spurs of female gonopods, the terminal claw of the female gonopods tridentate. It can however be distinguished from the latter by 5–6 ocelli versus 7 ocelli, Tömösváry’s organ smaller than adjoining ocellus versus larger than adjoining ocellus, 1222 coxal pores other than 3333 coxal pores, male legs 15 absence of secondary sexual characters on femur versus presence secondary sexual characters, ventral plectrotaxy 01320, dorsal plectrotaxy 10210 in the 14th legs, 01321 respectively 10310 in Lithobius (Monotarsobius) dziadoszi. The new species is morphologically close to Lithobius (Monotarsobius) riedeli Matic, 1970, from Korea, with which it shares the following traits: antennae composed of 15–19 articles, 2+2 coxosternal teeth, 2+2 spurs of female gonopods, the terminal claw of the female gonopods tridentate. It can however be distinguished from the latter by Tömösváry’s organ smaller than adjoining ocellus versus larger than adjoining ocellus, 1222 coxal pores other than 2222 or 3333 coxal pores, male legs 15 absence of secondary sexual characters on femur versus presence secondary sexual characters, ventral plectrotaxy 01320, dorsal plectrotaxy 10210 in the 14th legs, 01210 respectively 10200 in Lithobius (Monotarsobius) riedeli. The new species is morphologically close to Lithobius (Monotarsobius) mroczkowskii Matic, 1970, from Korea, with which it shares the following traits: 5–6 ocelli, 2+2 coxosternal teeth, 2+2 spurs of female gonopods, male legs 15 absence of secondary sexual characters on femur. It can however be distinguished from the latter by antennae composed of 15–19 articles versus 20–21 articles, 1222 coxal pores other than 3343 or 4564 coxal pores, the terminal claw of the female gonopods tridentate other than simple, ventral plectrotaxy 01320, dorsal plectrotaxy 10210 in the 14th legs, 01332 respectively 10311 in Lithobius (Monotarsobius) mroczkowskii.

**Table 1.** Leg plectrotaxy of Lithobius (Monotarsobius) zhangi sp. n.

Legs	Ventral					Dorsal				
	C	Tr	P	F	Ti	C	Tr	P	F	Ti
1	-	-	p	am	m	-	-	p	a	a
2	-	-	p	am	m	-	-	p	ap	a
3–9	-	-	-	am	m	-	-	p	ap	ap
10	-	-	-	am	m	-	-	p	p	ap
11	-	-	p	am	m	-	-	p	p	ap
12	-	-	p	am	m	-	-	mp	p	a
13	-	-	p	am	m	-	-	mp	p	p
14	-	m	amp	am	-	a	-	mp	p	-
15	-	m	amp	am	-	a	-	mp	-	-

## Key to the Chinese and Korean species of Lithobius (Monotarsobius)

To assist in the identification of the Chinese and Korean of Lithobius (Monotarsobius), the following key is offered. This key emphasizes characters that can be examined without high-magnification microscopy; moreover, these characters are specific to the taxa occurring in China and Korea.

**Table:** 

1	1111 coxal pores	**Lithobius (Monotarsobius) monoforaminis Ma, Pei, Wu, Lin, Gai, 2012**
–	At least 1222 coxal pores	**2**
2	4–6 coxal pores	**3**
–	At most 3 coxal pores.	**5**
3	8–11 ocelli on each side of cephalic fig **Lithobius (Monotarsobius) crassipes L. Koch, 1862**
–	5–6 ocelli on each side of cephalic fig	**4**
4	5555 coxal pores, 3+3, 4+4, 3+4 spurs of female gonopods	**Lithobius (Monotarsobius) ramulosus (Takakuwa, 1941)**
–	3343 or 4564 coxal pores, 2+2 spurs of female gonopods	**Lithobius (Monotarsobius) mroczkowskii Matic, 1970**
5	Four ocelli on each side of cephalic fig, 17+17 antennal articles	**Lithobius (Monotarsobius) crassus (Loksa, 1965)**
–	Five or more ocelli on each side of cephalic fig, antennal not less than 18+18 articles	**6**
6	Tömösváry’s organ smaller than adjoining ocellus	**7**
–	Tömösváry’s organ larger than adjoining ocellus or about same in size	**8**
7	With anterior spine on prefemur on legs 14–15	**Lithobius (Monotarsobius) zhangi sp. n.**
–	Without anterior spine on prefemur on legs 14–15	**Lithobius (Monotarsobius) songi Pei, Ma, Shi, Wu, Zhou, 2011**
8	male legs 15 presence secondary sexual characters, the terminal claw of the female gonopods tridentate	**9**
–	male legs 15 absence secondary sexual characters, the terminal claw of the female gonopods not tridentate	**10**
9	Ventral plectrotaxy 01210, dorsal plectrotaxy 10200 in the 14th legs	**Lithobius (Monotarsobius) riedeli Matic, 1970**
–	Ventral plectrotaxy 01321, dorsal plectrotaxy 10310 in the 14th legs	**Lithobius (Monotarsobius) dziadoszi Matic, 1970**
10	Tömösváry’s organ larger than the biggest ocellus	**Lithobius (Monotarsobius) holstii (Pocock, 1895)**
–	Tömösváry’s organ smaller than the biggest ocellus	**11**
11	With one protuberance at the end of the dorsal of tibia of 15 legs in male	**Lithobius (Monotarsobius) ferganensis (Trotzina, 1894)**
–	Without protuberance at the end of the dorsal of tibia of 15 legs in male	12
12	With posterior spine on prefemur on legs 11–13	**Lithobius (Monotarsobius) obtusus (Takakuwa, 1941)**
–	Without posterior spine on prefemur on legs 11–13	**Lithobius (Monotarsobius) subspinipes Ma, Pei, Zhu, Zhang, Liu, 2009**

## Supplementary Material

XML Treatment for Lithobius (Monotarsobius) zhangi
